# Personal social networks and organizational affiliation of South Asians in the United States

**DOI:** 10.1186/s12889-018-5128-z

**Published:** 2018-02-05

**Authors:** Namratha R. Kandula, Andrew J. Cooper, John A. Schneider, Kayo Fujimoto, Alka M. Kanaya, Linda Van Horn, Lawrence deKoning, Juned Siddique

**Affiliations:** 1 Feinberg School of Medicine, Division of General Internal Medicine, Northwestern University, Chicago, IL USA; 2 Feinberg School of Medicine, Department of Preventive Medicine, Northwestern University, Chicago, IL USA; 3 Department of Medicine and Public Health Sciences and the Chicago Center for HIV Elimination, University of Chicago, Chicago, IL USA; 4 Division of Health Promotion & Behavioral Sciences, School of Public Health, University of Texas Health Science Center at Houston, Houston, TX USA; 5 Division of General Internal Medicine, University of California San Francisco, San Francisco, CA USA; 6 Department of Pathology, University of Calgary, Calgary, Alberta Canada; 7 Department of Laboratory Medicine, Pediatrics, University of Calgary, Calgary, Alberta Canada; 8 Department of Community Health Sciences, University of Calgary, Calgary, Alberta Canada; 9 Northwestern University, 420 E. Superior Street, 6th Floor, Chicago, IL 60611 USA

**Keywords:** Asian American, Health, Social support, Social networks, Self-rated health, Health-related decision making

## Abstract

**Background:**

Understanding the social lives of South Asian immigrants in the United States (U.S) and their influence on health can inform interpersonal and community-level health interventions for this growing community. This paper describe the rationale, survey design, measurement, and network properties of 700 South Asian individuals in the Mediators of Atherosclerosis in South Asians Living in America (MASALA) social networks ancillary study.

**Methods:**

MASALA is a community-based cohort, established in 2010, to understand risk factors for cardiovascular disease among South Asians living in the U.S. Survey data collection on personal social networks occurred between 2014 and 2017. Network measurements included size, composition, density, and organizational affiliations. Data on participants’ self-rated health and social support functions and health-related discussions among network members were also collected.

**Results:**

Participants’ age ranged from 44 to 84 (average 59 years), and 57% were men. South Asians had large (size=5.6, SD=2.6), kin-centered (proportion kin=0.71, SD=0.28), and dense networks. Affiliation with religious and spiritual organizations was perceived as beneficial to health. Emotional closeness with network members was positively associated with participants’ self-rated health (*p*-value <0.001), and networks with higher density and more kin were significantly associated with health-related discussions.

**Discussion:**

The MASALA networks study advances research on the cultural patterning of social relationships and sources of social support in South Asians living in the U.S. Future analyses will examine how personal social networks and organizational affiliations influence South Asians’ health behaviors and outcomes.

**Trial registration:**

ClinicalTrials.gov identifier: NCT02268513

**Electronic supplementary material:**

The online version of this article (10.1186/s12889-018-5128-z) contains supplementary material, which is available to authorized users.

## Background

South Asians (individuals from India, Pakistan, Bangladesh, Sri Lanka, Nepal, Bhutan, and Maldives) are the second fastest growing racial/ethnic group in the United States (U.S.), after Latinos [1]. South Asians have an elevated risk of cardiovascular disease (CVD) and diabetes mellitus, and a higher ischemic heart disease mortality rate than non-Latino Whites and other Asians [2–6]. Despite a growing body of research to explain and reduce these disparities, individual health behavior and clinical risk factors do not fully explain South Asians elevated cardiometabolic risk [3, 7]; further, individual level prevention interventions have had limited success in this high risk group ([8–10]). Thus, widening inquiry beyond the individual, to the larger social drivers of health,[11] may offer key insights into the less-well documented social context of health outcomes in at-risk communities.

Prior research suggests that social relationships exert an especially important influence on behaviors and beliefs of South Asians in the U.S. [12–15]. Almost 90% of U.S. South Asians are first generation immigrants who believe that kinship and family ties are paramount, with an emphasis on collectivism, social control, and maintenance of group identity [12–15]. Studies show that South Asians have low levels of physical activity and dietary patterns that contribute to increased cardiometabolic risk [3, 16, 17]. These behaviors are socially and culturally informed [18, 19]; yet there is limited understanding of the structure, composition, and function of social network ties among South Asians, the cultural patterning of networks, and how social relationships influence the health of this community. Understanding South Asians’ social lives, their specific functions, and how they are linked to health can help inform effective interpersonal and community-level health interventions for the rapidly growing South Asian community.

Social networks influence health via many mechanisms including: social influence and control; establishment of health beliefs and normative behaviors; feelings of shared identity and belonging; access to resources; and provision of support [20, 21]. Social networks transmit information, attitudes, and behaviors that determine health outcomes [20]; early understandings of network processes suggested mechanisms of network influence through social diffusion such that new ideas and behaviors are spread through contact with other people who have already adopted the behavior [22].

In addition, voluntary affiliation and participation in a community, religious, or social organization has also been shown to influence network composition, social support, and health [23]. Voluntary membership in organizations has the potential to create more or less diverse social connections and exposure to additional sources of social influence, norms, and support. Thus, measurement of both personal social networks and organizational affiliation may provide novel insights into the social influence processes relevant to health in South Asians.

The overall goal of this research project was to investigate both personal social networks and organizational affiliation in South Asian community, religious, or social organizations among individuals who participated in the Mediators of Atherosclerosis in South Asians Living in America (MASALA) study, a prospective community-based cohort study on CVD risk and incidence in the U.S. South Asian population [24]. Although a body of research has shown that social networks are associated with health behaviors and outcomes, there is almost nothing known about the personal social networks and organizational affiliations of South Asian immigrants in the U.S. [25, 26]. This study provides the unique opportunity to advance research on the cultural patterning of social connections in South Asian immigrants and begins to explore the linkages between social networks and health.

## Methods

### Participants

The Mediators of Atherosclerosis in South Asians Living in America (MASALA) Study is a community-based cohort of South Asians who were free from CVD at baseline. Details regarding recruitment and baseline measurements have been published previously [24]. Briefly, using surname-based recruitment methods, a community-based sample of 906 South Asians (age range: 40-84 y, 46% women, 98% foreign-born) was recruited between October 2010 and March 2013 from the nine counties of the San Francisco Bay Area and seven census tracts close to Chicago, IL and surrounding suburbs. To be eligible for the baseline MASALA exam, participants had to self-report South Asian ethnicity, be between the ages of 40-84 years inclusive, and be able to speak and/or read English, Hindi or Urdu. Exclusion criteria included a physician diagnosed heart attack, stroke or transient ischemic attack, heart failure, angina, use of nitroglycerin, a history of cardiovascular procedures, current atrial fibrillation, active treatment for cancer, life expectancy < 5 years due to a serious medical illness, impaired cognitive ability, plans to move out of the study region in the next 5 years, living in a nursing home or on a waiting list, and weight > 300 lbs.

### Ethics, consent and permissions

The study protocol and procedures were approved by two institutional review boards and all study participants signed informed consent.

### Measurement of personal social networks

From 2014-2017, the MASALA study participants were re-enrolled for a 2^nd^ study visit where personal network characteristics were measured using a standard egocentric approach that examined the network members (alters) reported by the respondent (ego). The surveys were administered in English, Hindi, or Urdu by trained interviewers. To collect egocentric network data on respondents’ close confidantes, interviewers asked respondents to enumerate relevant alters by using a name generator that has been used by the the General Social Survey [27] (years 1985 and 2004) and the National Social Life, Health, and Aging Project’s (NSHAP) social networks module [28] to collect data on participants’ core confidants. Interviewers asked participants to list the people with whom they discuss “important matters.” Respondents could name up to ten people; this name generator was selected to identify network “confidants” who have opportunities to exert social influence and normative pressure [20, 29]. Studies using this approach have yielded important insights about social contacts who are particularly influential [30–32].

Following the enumeration of alters, the interviewers continued with *name interpreter* questions, which were used to collect information about the first five network members who were listed. Limiting responses to five individuals is a typical approach to reduce respondent burden and the first alters names typically represent the most important individuals within the social network. Name interpreters helped to characterise the type of relationships (e.g., spouse or significant other, friend), sociodemographic characteristics (e.g. age, country of birth), strength of relationship (emotional closeness, frequency of contact), functions of the network members (e.g., social support), discussion topics (e.g., health) and frequency of communication among the five alters.

### Measurement of organizational affiliation

South Asians in the U.S. have developed organizational structures that may exert a strong influence on social connections, social support, and cultural beliefs related to health behaviors. We developed a roster of South Asian organizations (religious, social, cultural, community-based organizations) in Chicago and the San Francisco Bay area using key informant input and an iterative approach. Respondents were asked to look through the pre-defined list and circle the organization(s) that they visited within the prior 12 months and to also circle how frequently they visited the organization in the prior 12 months. Respondents could choose multiple organizations if applicable, and they were also given the option to add an organization if it was not on the roster. There was no limit on the number of organizations a person could affiliate with. However, after reporting all their organizational ties, respondents were only asked in more detail about the 6 places they attended most frequently. The study team limited additional responses to the 6 organizations visited most frequently to reduce participant burden and because the organizations visited most frequently were likely to have the greatest influence.

After data collection, the organizations were coded as community-based organizations, spiritual organizations, and places of worship (i.e. temples, churches, and mosques). We used the Internal Revenue Service definition for coding these and distinguishing spiritual organizations from places of organized worship (https://www.irs.gov/charities-non-profits/churches-religious-organizations). Spiritual organizations focused on religious and spiritual teaching, but were non-denominational, not considered places of worship, and did not provide organized religious services like a church, temple, or mosque.

### Social network measures

The MASALA network data are provided in a dyad-level file in which each row contains information about a specific network member for a given respondent (i.e., multiple rows per respondent). The most basic measure of personal network structure was size*:* the number of names mentioned in response to the first name generator question. The remaining network variables were calculated using information on the first five individuals listed in response to the name generator question.

Density was defined as the number of ties divided by the number of pairs [33]. A tie was defined as whether or not there was any reported communication between two alters. A fully dense network (d=1.0) indicates that all network members were connected to each other. Densely connected networks typically have a great deal of influence on an individual's behavior but may not offer access to new information or resources, whereas sparsely connected networks may allow for the introduction of new information, but may provide less tangible support [34].

Network composition variables, which examine characteristics of alters, included the proportion of specific characteristics: South Asian origin, household member, kin, and gender; in a MASALA respondent’s network. We also calculated the average closeness rating (1(low) to 5 (high)) across alters and the volume of contact with alters as contact-days/year based on the participants’ reports of how often they talked to each alter on a 5-point scale, ranging from every day to a few times a year. We calculated the average number of organizational affiliations for each participant.

### Assessing social support

The interview asked respondents about emotional social support (e.g. “How often can you share your worries?”) and instrumental social support (e.g. “How often can you rely on this person for help?”) received from the five alters. Responses were categorized as “most of the time,” “sometimes,” and “rarely/never.” We also asked respondents to report on negative social interactions with each alter, such as, “how often does [alter name] make too many emotional or physical demands on you,” and “how often does [alter name] criticize you?”

### Health outcomes

Self-rated health was measured by asking participants to rate their health on a continuous scale of 1–10, with 1 being poor health and 10 being excellent health. A categorical measure of self-rated health (excellent, very good, good, fair, and poor) was avoided given there is a wide range of variability in how those of ethnic minority status and foreign born perceive these categories of health [35].

For each alter listed, the interviewer asked, “Suppose you had a health problem that you were concerned about, or needed to make an important decision about your own medical treatment. How likely is it that you would talk with [name] about this: Would you say very likely, somewhat likely, or not likely”? This question was the same as what was used in NSHAP. We calculated the proportion of the network that the participant was ‘very likely’ to talk with about health.

### Respondent sociodemographics and cultural characteristics

Information on participants’ education, income, age, marital status, birthplace, number of years living in the U.S., and religion were collected as previously described [24]. Cultural characteristics were captured using multiple items. The traditional beliefs scale was a continuous measure asking participants how much they wished South Asian cultural traditions would be practiced in the U.S. Examples of these cultural traditions centered upon food related activities (fasting, eating traditional South Asian foods like chapattis and daal) and partaking in arranged marriage practices [36]. The scale had a Cronbach’s alpha coefficient of .81 and ranged from 0 to 28 with lower scores reflecting stronger cultural beliefs and higher scores reflecting weaker cultural beliefs. We also asked participants about cultural self-identity by asking them to report on a scale of 1(not at all)-10 (extremely), “How South Asian do you feel,” and “How American do you feel?”

### Statistical analysis

We calculated descriptive statistics for all variables of interest, including participant characteristics, network characteristics, alter relationships, alter characteristics, and organizational ties. We examined bivariate associations between participant and network characteristics using Pearson’s correlations and tested whether these correlations were significantly different from 0. Network variables were modeled as continous variables. For presentation in tables, we categorized some continuous participant characteristics (e.g. age, traditional cultural beliefs, education) because it allowed us to clearly (and parsimoniously) describe how network characteristics may differ as a function of participant characteristics. However, when calculating correlations, participant characteristics were analyzed on their original (continuous) scale in order to better preserve relationships between participant characteristics and network characteristics and also to avoid the loss of statistical power that would be the result of collapsing continuous data into discrete categories.

We described alter social support and negative social interactions by their relationship to the ego. We also described participants’ organizational affiliation by organization type and attendance at health-related events at these organizations.

Lastly, we used adjusted linear regression models to examine if network density, closeness with alters, network composition variables, and number of organization affiliations were associated with self-rated health or the proportion of the network with which the ego was “very likely” to discuss his/her health. Each network characteristic was included as a predictor in separate regression models adjusted for age, sex, education, and network size.

All statistical tests were performed using two-sided tests with *α* = 0.05 and were conducted using SAS, version 9.4 (SAS Institute; Cary, NC).

## Results

### Participant characteristics

The MASALA study participants who completed the social network module (*n*=700) were on average 59 years old (SD=9), 43% were women, and 90% were married or with a partner (Table 1). Overall, participants had high income levels and high education with 88% having at least a bachelor’s degree. The majority of MASALA participants were born outside the U.S. (98%), and 65% had been living in the U.S. for more than 25 years. Participants’ mean self-rated health was 7.9 (SD=1.4) and ranged from 3 to 10.

**Table 1:** Characteristics of participants from the Mediators of Atherosclerosis in South Asians Living in America (MASALA) study, who participated in the social networks survey, 2014-2017

Characteristic	*N*=700 (SD)	
Age, years; Mean (SD)	59.0	9.1
Age, years, N (%)		
44-49	122	17.4
50-59	257	36.7
60-69	212	30.3
70-84	109	15.6
Female, N (%)	300	42.9
Self-rated health, Mean (SD)	7.6	1.4
Education, N (%)		
< Bachelor's degree	78	11.1
= Bachelor's degree	197	28.1
> Bachelor's degree	425	60.7
Income, N (%)		
< $75k	165	23.6
$75k to < $100 k	68	9.7
>= $100 k	447	63.9
Unknown	20	2.9
Marital status, N (%)		
Married/living as married/living with partner	630	90.0
Years Living in U.S., N (%)		
<15 years	51	7.3
15-25 years	191	27.3
>25 years	458	65.4
Birthplace, N (%)		
Bangladesh	3	0.4
India	591	84.4
Nepal	1	0.1
Pakistan	26	3.8
Sri Lanka	8	1.1
United States	17	2.4
Other	54	7.7
Religion (not mutually excl.), N (%)		
Buddhism	6	0.9
Christianity	28	4.0
Hinduism	483	69.0
Islam	41	5.9
Jainism	50	7.1
Sikhism	59	8.4
Zoroastrianism	2	0.3
None	45	6.4

### Network size and composition

Among the 700 participants, there were a total of 2,932 network members identified. All participants reported at least one confidant, and the average network size was 6 (SD=3) (Table 2). Over two thirds of the sample reported that they had at least five or more confidants.

**Table 2:** Bivariate association of participant characteristics with network structure and composition, the Mediators of Atherosclerosis in South Asians Living in America (MASALA) social networks study, 2014-2017

	N	Network Size	Proportion Kin	Prop South Asian	Prop Female	Prop living in same house	Closeness to alters (mean; 1 low - 5 high)	Volume of contact with alters (contact-days/year)	Network density	Number of organizational affiliations
Overall	700	5.6	0.71	0.88	0.55	0.32	4.4	1069	0.78	4.7
*SD*		2.6	0.28	0.24	0.25	0.25	0.5	390	0.26	4.3
Age, years (SN visit)										
40-49	122	5.8	0.64	0.83	0.53	0.37	4.5	1130	0.78	4.1
50-59	257	5.7	0.69	0.87	0.57	0.32	4.4	1093	0.75	4.4
60-69	212	5.3	0.75	0.90	0.55	0.31	4.5	1047	0.80	4.8
70+	109	5.7	0.79	0.90	0.52	0.26	4.5	989	0.81	5.8
*P*-value		*p*=0.52	*p* <0.01	*p*< 0.01	*p*=0.83	*p*<0.01	*p*=0.22	*p*<0.01	*p*<0.05	*p*<0.01
Gender										
Male	400	5.4	0.71	0.87	0.48	0.34	4.4	1031	0.79	4.8
Female	300	5.9	0.72	0.89	0.64	0.29	4.5	1120	0.77	4.5
*P*-value		*p* <0.05	*p*=0.93	*p*=0.44	*p*<0.01	*p*=0.01	*p*=0.01	*p*<0.01	*p*=0.21	*p*=0.37
Education										
< Bachelor's degree	78	4.7	0.80	0.91	0.57	0.38	4.4	1109	0.86	4.7
= Bachelor's degree	197	5.3	0.70	0.91	0.56	0.33	4.5	1057	0.75	4.8
> Bachelor's degree	425	5.9	0.70	0.86	0.54	0.30	4.5	1068	0.78	4.6
*P*-value		*p*<0.01	*p* <0.05	*p*<0.01	*p*=0.16	*p*<0.01	*p*=0.44	*p*=0.60	*p*=0.30	*p*=0.65
Income										
< $75k	165	4.8	0.76	0.91	0.55	0.34	4.5	1018	0.80	5.2
$75k to < $100k	68	5.5	0.73	0.86	0.56	0.28	4.4	1043	0.76	4.8
>= $100k	447	6.0	0.69	0.86	0.55	0.31	4.4	1096	0.78	4.5
*P*-value		*p*<0.01	*p*<0.01	*p* <0.05	*p*=0.85	*p*=0.17	*p*=0.92	*p* <0.05	*p*=0.42	*p*=0.10
Sum of Traditional Cultural Beliefs										
Tertile 1: 0 to 11 (Most traditional)	231	5.3	0.73	0.91	0.53	0.33	4.5	1075	0.79	5.8
Tertile 2: 12 to 16 (Some traditional)	218	5.9	0.73	0.90	0.54	0.33	4.5	1129	0.82	4.9
Tertile 3: 17 to 28 (Least traditional)	250	5.6	0.69	0.83	0.57	0.29	4.4	1013	0.75	3.4
P-value		*p*=0.21	*p*<0.10	*p*<0.01	*p*<0.10	*p*=0.11	*p*=0.25	*p*<0.10	*p*=0.17	*p*<0.01
How South Asian do you feel?										
1-5 (Low)	98	5.4	0.67	0.75	0.58	0.32	4.4	961	0.75	3.1
6-10 (High)	602	5.7	0.72	0.90	0.54	0.32	4.4	1087	0.79	4.9
*P*-value		*p*=0.80	*p* <0.05	*p*<0.01	*p* <0.10	*p*=0.33	*p*<0.01	*p*<0.01	*p*=0.05	*p*<0.01
How American do you feel?										
1-5 (Low)	377	5.5	0.74	0.92	0.56	0.33	4.4	1102	0.80	4.8
6-10 (High)	323	5.7	0.68	0.83	0.54	0.30	4.5	1031	0.76	4.5
*P*-value		*p*=0.22	*p*<0.01	*p*< 0.01	*p*=0.31	*p* <0.10	*p*=0.18	*p* <0.10	*p*=0.24	*p*=0.41
Marital status										
Married/living as married/living with partner	630	5.6	0.73	0.89	0.54	0.34	4.5	1084	0.80	4.7
Single/separated/widowed/divorced	70	5.4	0.58	0.81	0. 66	0.11	4.3	937	0.62	4.3
*P*-value		*p*=0.39	*p*< 0.01	*p*= 0.01	*p*< 0.01	*p*< 0.01	*p*<0.01	*p*<0.01	*p*<0.01	*p*=0.44

Note: Continuous participant characteristics (i.e. age, income, traditional cultural beliefs, how South Asian do you feel, how American do you feel) have been categorized for presentation purposes. *P*-values of statistical tests for these participant characteristics are based on correlation coefficients which treat these variables as continuous.

The correlation between network variables is shown in Additional file 1: Table S1.

Overall, South Asians’ personal networks were mostly comprised of kin (proportion of kin 0.71) and the majority of network members were South Asian (proportion South Asian=0.88). There were, however, some significant differences by sociodemographic characteristics and cultural identity. Compared to the youngest age group (44-49 years), older South Asians had social networks that were significantly more kin-centered and more South Asian. Older South Asians were also affiliated with more South Asian organizations than those in the youngest age group. Education and income were also associated with different social network compositions (Table 2); individuals in the highest education and income categories had significantly larger networks that had a lower proportion of kin compared to those with less education.

Cultural beliefs and identity were also significantly associated with network composition; individuals with stronger traditional South Asian cultural beliefs had more ethnically homogenous networks (proportion South Asian= 0.91 and 0.90, respectively) than South Asians with weaker traditional beliefs (proportion South Asian=0.83). A stronger South Asian identity was positively and significantly associated with network kin proportion, proportion South Asian, and the number of affiliations with South Asian organizations.

### Emotional closeness and volume of contact

South Asians reported being emotionally very close to their confidants (average closeness=4.4, SD=0.5), and on average, respondents reported three contacts per day with a close confidant. Egos said they mostly communicated with over half (53%) of their network in-person, and with 41% by telephone. Women, married individuals, and respondents with a stronger South Asian identity reported significantly more contact with their network members. As age increased, South Asians had a significantly lower volume of contact per year with network members.

### Network density

Network density is a measure of all possible ties that existed between alters. Overall, South Asians had high density networks, with 78% of all possible ties among alters being present (Table 2). Older individuals, less educated respondents, and those who were married/living with a partner had significantly more dense networks.

### Social support and negative social interactions

Participants reported lower levels of emotional support (i.e., opening up about worries) than instrumental support (i.e., being able to rely on someone when you have a problem) from their network members. South Asians said they could talk about their worries most of the time with 54% of all network members (Table 3), with spouses/partners being the most common source of emotional support. Participants said they could rely on 94% of spouses/partners, 80% of children, and two-thirds of siblings, friends or other kin most of the time if they had a problem and needed help. Negative social interactions were more common with spouses/partners and children than other types of alters. Across all networks, 52% of spouses/partners and 33% of children were described as making too many emotional, physical, or psychological demands sometimes/most of the time (Table 3), and 61% and 38%, respectively, were described as criticizing the ego.

**Table 3:** Social support and negative social interactions by alter type, the Mediators of Atherosclerosis in South Asians Living in America (MASALA) social networks study, 2014-2017

	Alter Relationship													
	Spouse/Partner		Child		Sibling		Other Kin		Friend		Other		Total	
	N	%	N	%	N	%	N	%	N	%	N	%	N	%
Talk about worries														
Most of the time	439	77.7	388	54.3	186	49.3	182	41.8	364	51.1	47	25.0	1606	53.7
Sometimes	104	18.4	241	33.7	153	40.6	162	37.2	225	31.6	66	35.1	951	31.8
Rarely/Never	22	3.9	86	12.0	38	10.1	91	20.9	124	17.4	75	39.9	436	14.6
Rely on														
Most of the time	530	93.8	575	80.4	259	68.7	294	67.6	487	68.3	91	48.4	2236	74.7
Sometimes	30	5.3	92	12.9	86	22.8	85	19.5	158	22.2	61	32.4	512	17.1
Rarely/Never	5	0.9	48	6.7	32	8.5	56	12.9	68	9.5	36	19.1	245	8.2
Makes too many demands														
Most of the time	94	16.6	51	7.1	10	2.7	9	2.1	6	0.8	9	4.8	179	6.0
Sometimes	199	35.2	188	26.3	66	17.5	76	17.5	62	8.7	24	12.8	615	20.5
Rarely/Never	272	48.1	476	66.6	301	79.8	350	80.5	645	90.5	155	82.4	2199	73.5
Criticizes you														
Most of the time	87	15.4	37	5.2	9	2.4	4	0.9	11	1.5	2	1.1	150	5.0
Sometimes	258	45.7	236	33.0	105	27.9	69	15.9	97	13.6	34	18.1	799	26.7
Rarely/Never	220	38.9	442	61.8	263	69.8	362	83.2	605	84.9	152	80.9	2044	68.3
Total	565	100	715	100	377	100	435	100	713	100	188	100	2993	100

### Organizational ties

The 700 participants in this study reported a total of 3,213 organizational ties (average number of ties per participants=5, SD=4). The majority of organizational ties were with places of worship (Table 4). Among the 2,411 organizations that participants visited most frequently, over 51% of these ties were long standing (>10 years), and participants reported feeling close to 67% of the organizations. Attendance at these organizations was perceived as beneficial to health, ranging from 68% of community based organizations to 88% of mosques being perceived as beneficial. Participants reported talking with other people at 80% of organizations they attended, but few attended any health-related events at these places.

**Table 4:** Participants’ affiliation with south Asian organizations, the Mediators of Atherosclerosis in South Asians Living in America (MASALA) social networks study, 2014-2017

	Overall		Community-Based Organizations (CBO)		Spiritual Organizations		Churches		Temples		Mosques	
	N	%	N	%	N	%	N	%	N	%	N	%
Affiliations (participant/organization pairs)	2411	-	643	-	86	-	31	-	1576	-	75	-
Attended >10 years	1235	51.2	302	47.0	37	43.0	13	41.9	835	53.0	48	64.0
Feel very/somewhat close to organization	1619	67.2	388	60.3	66	76.7	24	77.4	1086	68.9	55	73.3
Attendance improves health*	1776	73.7	435	67.7	73	84.9	25	80.6	1177	74.7	66	88.0
Eat food at organization**	1498	62.1	383	59.6	23	26.7	20	64.5	1035	65.7	37	49.3
Attend health screening at organization**	98	4.1	31	4.8	3	3.5	1	3.2	53	3.4	10	13.3
Attend healthy eating or exercise activity at organization**	99	4.1	42	6.5	14	16.3	1	3.2	40	2.5	2	2.7
Talk with other people at organization**	1926	79.9	581	90.4	73	84.9	30	96.8	1176	74.6	66	88.0

Note: Spiritual organizations focused on religious or spiritual teaching, but were non-denominational, not recognized as places of worship, and did not provide organized religious services like a church, temple, or mosque.

*Indicates participants who reported, “A little bit, somewhat, quite a bit or very much” versus “Not at all”

**Indicates participants who reported, “Sometimes, usually or always” versus “Never”

### Association of social network characteristics with health outcomes

In unadjusted and adjusted linear regression models, only closeness to alters was significantly associated with self-rated health; as closeness with alters increased, self-rated health increased (adjusted β=0.27, 95% CI= 0.07, 0.48) (Data not shown). Several network characteristics were associated with the proportion of the network that the ego was very likely to discuss his/health with (Table 5). Overall, the proportion of network members that participants were very likely to discuss a health problem with was 0.65 (SD=0.30). Network density and closeness to alters were each positively associated with the network proportion involved in health discussions (adjusted β=0.29 95% CI=0.21, 0.37 and adjusted β=0.17, 95% CI=0.13, 0.21. respectively). A higher proportion of kin and a higher proportion of South Asians were also significantly associated with a higher proportion of the network involved in health discussions. The proportion of women in the network, volume of contact with alters, and number of organizational affiliations were not associated with health discussions.

**Table 5:** Regression analyses of network characteristics and health discussions, the Mediators of Atherosclerosis in South Asians Living in America (MASALA) social networks study, 2014-2017

Proportion of Network that Ego Discusses Health With		
Network characteristic	Beta-coefficient	95% CI
Network Density	0.29	0.21, 0.37 ***
Closeness to alters	0.17	0.13, 21 ***
Proportion Kin	0.30	0.22, 38 ***
Prop South Asian	0.14	0.04, 23 **
Prop Female	-0.06	-0.16, 0.03
Prop living in same house	0.23	0.13, 0.32 ***
Volume of contact with alters (contact-days/year)	<0.01	<-0.01, <.01
Number of organizational affiliations	<0.01	<-0.01, 01

Note: Each network variable regressed separately on outcome and adjusted for age, sex, education, and network size. All analyses included *n*=700, except for network density which was *n*=684 because of missing data.

* *p* < .05; ** *p* < .01; *** *p* < .001

## Discussion

The MASALA social networks ancillary study is the first comprehesive profile of middle- and older-aged South Asian adults’ social networks and association of network characteristics and functions with health. We found that South Asians living in the U.S. have a relatively large confidant network, which is mainly kin-centered and comprised of individuals who are also South Asian. Network characteristics, including size, composition, and density varied by participants’ age, sex, education, income, and cultural factors, suggesting potentially important subgroup differences in social context, which in turn effects sources of influence and support, as well as types of information and resources available to South Asian immigrants. We also found that networks that were more dense, emotionally closer, and had a higher proportion of kin and South Asians, were positively associated with health-related discussions.

Until now, there have been no data on social networks and health in U.S. South Asians, and less than a handful of studies on South Asians in India and the United Kingdom. We found that South Asians reported larger confidant networks (size=5.6, SD=2.6) compared to prior studies in the U.S. and India; however, previous network studies may not have captured the full extent of the personal network because they placed a smaller limit (maximum of 5) on the number of alters reported during the name generator. The present study used a more open-ended approach and allowed respondents to name up to 10 people during the name generator; our study may be more reflective of true network size. [26, 28, 37] Similar to Latino immigrants in the U.S.[37], South Asian immigrants appeared to have dense, kin-centered networks that were ethnically homogenous. Eighty percent of network ties among urban Asian Indians were family members [26]; although this is slightly higher than what we found, both studies demonstrate that family relationships are central to South Asian social networks. Interestingly, in our study, several social and cultural factors were associated with the proportion kin. Higher education and income and a stronger American identity and weaker South Asian identity were associated with a significantly lower proportion of kin in the network, suggesting that socioeconomic status and cultural change influence social network composition. Others have also shown that lower socioeconomic status is associated with a higher proportion of kin in U.S. populations [33, 38].

We also examined different types social support and interactions among South Asians and found that family members were most common sources of emotional and instrumental support. Interestingly, South Asians appeared to report less emotional support (being able to talk about worries) from network members, including family, than instrumental (being able to rely on when there is a problem). However, the questions used in our survey may not have distingushed between the “availability” of support and “seeking” support; this may be an important distinction since some cross-cultural studies have indicated that Asian Americans tend to seek less support than other racial/ethnic groups [39]. Our findings deserve further exploration to determine if there are differences in willingness of South Asians to share emotional concerns and seek emotional support compared to other types of social support. Future analyses will examine if there are links between network characteristics, types of social support, and health in South Asians.

The findings that weaker South Asian ethnic identity and weaker traditional cultural beliefs were associated with networks that were less ethnically homogenous, and less dense may have important implications for the norms, health information, and resources available to South Asians. Others have found that ethnocultural identity impacted peer group choices and was associated with personal network composition in immigrant adolescents [40, 41]. As a next step, we will examine if these network differences are associated with differences in social norms, influence, and support among South Asians immigrants, which in turn could influence health and behavior.

We also found that South Asian immigrants associate with and attend a large number of local South Asian organizations, including community, social, and religious institututions. Participants perceived attendance at these organizations as beneficial to their health and reported socializing with other members, suggesting that South Asian organizations may provide additional sources of support and social connections. In addition, co-participation in South Asian organizations also provides the opportunity for exisiting norms and behaviors to be reinforced. As a next step, we will examine if attendance and affiliation with these organizations influences health behaviors and outcomes [29], and if co-participation by MASALA study participants in specific organizations is associated with diet, exercise, and obesity [42]. Our findings are a starting point for determining if South Asian community structures can be leveraged for health interventions. Religious and spiritual organizations were the most commonly reported affiliations, and the potential of these organizations for health promotion and intervention should be explored.

Others have found associations between social networks and self-rated health [43, 44], with smaller networks being associated with worse health in the elderly and larger, more family-based networks being associated with better self-reported health. In our study, we only found that greater emotional closeness with alters associated with better self-rated health [21], but did not find associations with network size or proportion kin. Close alters may provide higher levels of social support or access to other resources that improve perceived health. We also showed that network structure and composition, including density, closeness, and proportion kin, were associated with health discussions among South Asians and their network members. Our data lend additional empiric support to prior studies showing that South Asian immigrants rely on close family members for health information and advice [45]. How these health discussions influence behaviors or health outcomes is an area that has yet to be explored. It would also be intersting to investigate if specific health issues (mental health, domestic violence, sexually transmitted diseases) are as likely to be discussed in South Asian families as general health problems.

Although an egocentric study provides a feasible way of obtaining network information on a large-scale, it has several limitations. Egocentric data is based purely upon the knowledge, reflection, and recall of the ego, which may be inaccurate – especially when describing the relationship between two alters [46]. Because we did not observe alter’s view of relationships, we were not able to validate the ego self-report. The analysis is still relevant, however, if we consider the fact that an ego’s perception of relationships may be more important than whether or not the perceived relationship is validated by the alter [47]. We also did not ask participants about affiliations with non-South Asian organizations or about organizations outside their state of residence, thus limiting our understanding of the full range of potential organizational associations South Asians may have, however, the organizations in the roster represent the major organizations in the lives of South Asians In addition, because of the cross-sectional study design, causality cannot be inferred. Lastly, the MASALA study cohort includes middle- and older-aged South Asians, the majority of whom are Asian Indian immigrants with high socieconomic status. Importantly, our response rate to the social networks module was 78%, and non-responders were more likely to have low socieconomic status and be women.

While the sociodemographics of the MASALA cohort are similar to that of the general U.S. Asian Indian population [48], these results may not be generalizable to all South Asians. In particular, the social networks of U.S.-born South Asians may be quite different from those who immigrated as adults.

## Conclusions

People’s social lives shape their beliefs, norms, and availability of social support, all of which influence behavior and health. This is the first study to describe the structure and composition of U.S. South Asians’ personal social networks and organizational affiliation and provides new insight into the patterning of social relationships in this growing community. The MASALA networks data will be used to examine how participants’ attributes, network members’ characteristics, organizational membership, and the nature of their relationships with each other contribute to diet, exercise, weight, and cardiovascular health outcomes. This study provides a unique opportunity to investigate the connections between health and social context and to inform network-based health interventions to improve the health and well-being of U.S. South Asians.

## Additional file

Additional file 1: Table S1. Pairwise correlations of network variables, the Mediators of Atherosclerosis in South Asians Living in America (MASALA) Social Networks Study, 2014-2017. (DOCX 24 kb)

## Abbreviations

CI: Confidence Interval
*CVD*: Cardiovascular disease
MASALA: Mediators of Atherosclerosis in South Asians Living in America
NSHAP: National Social Life, Health, and Aging Project
*SD*: Standard deviation
U.S: United States of America

## References

[1] South Asian Americans Leading Together: A Demographic Snapshot of South Asians in the United States. http://saalt.org/wp-content/uploads/2012/09/Demographic-Snapshot-Asian-American-Foundation-2012.pdf In*.*; Accessed on 2 June 2015.

[2] Bhopal R, Fischbacher C, Vartiainen E, Unwin N, White M, Alberti G (2005). Predicted and observed cardiovascular disease in South Asians: application of FINRISK, Framingham and SCORE models to Newcastle Heart Project data. Journal of public health.

[3] Joshi P, Islam S, Pais P, Reddy S, Dorairaj P, Kazmi K, Pandey MR, Haque S, Mendis S, Rangarajan S (2007). Risk factors for early myocardial infarction in South Asians compared with individuals in other countries. JAMA : the journal of the American Medical Association.

[4] McKeigue PM, Ferrie JE, Pierpoint T, Marmot MG (1993). Association of early-onset coronary heart disease in South Asian men with glucose intolerance and hyperinsulinemia. Circulation.

[5] McKeigue PM, Marmot MG (1988). Mortality from coronary heart disease in Asian communities in London. Bmj.

[6] Palaniappan L, Wang Y, Fortmann SP (2004). Coronary heart disease mortality for six ethnic groups in California, 1990-2000. Ann Epidemiol.

[7] Yusuf S, Joseph P (2017). The epidemic of cardiovascular disease in South Asians: Time for action. American Heart Journal.

[8] Ramachandran A, Snehalatha C, Mary S, Mukesh B, Bhaskar AD, Vijay V, Indian Diabetes Prevention P (2006). The Indian Diabetes Prevention Programme shows that lifestyle modification and metformin prevent type 2 diabetes in Asian Indian subjects with impaired glucose tolerance (IDPP-1). Diabetologia.

[9] Weber MB, Ranjani H, Staimez LR, Anjana RM, Ali MK, Narayan KM, Mohan V (2016). The Stepwise Approach to Diabetes Prevention: Results From the D-CLIP Randomized Controlled Trial. Diabetes Care.

[10] Kandula NR, Dave S, De Chavez PJ, Bharucha H, Patel Y, Seguil P, Kumar S, Baker DW, Spring B, Siddique J (2015). Translating a heart disease lifestyle intervention into the community: the South Asian Heart Lifestyle Intervention (SAHELI) study; a randomized control trial. BMC public health.

[11] Auerbach JD, Parkhurst JO, Caceres CF (2011). Addressing social drivers of HIV/AIDS for the long-term response: conceptual and methodological considerations. Glob Public Health.

[12] Watt L, Dix D, Gulati S, Sung L, Klaassen RJ, Shaw NT, Klassen AF. Family-centred care: a qualitative study of Chinese and South Asian immigrant parents' experiences of care in paediatric oncology. Child Care Health Dev. 2011; 10.1111/j.1365-2214.2011.01342.x 22066491

[13] van Esch SC, Cornel MC, Geelhoed-Duijvestijn PH, Snoek FJ (2012). Family communication as strategy in diabetes prevention: An observational study in families with Dutch and Surinamese South-Asian ancestry. Patient Educ Couns.

[14] Grewal S, Bottorff JL, Hilton BA (2005). The influence of family on immigrant South Asian women's health. J Fam Nurs.

[15] Sharma RK, Khosla N, Tulsky JA, Carrese JA (2012). Traditional expectations versus US realities: first- and second-generation Asian Indian perspectives on end-of-life care. J Gen Intern Med.

[16] Yusuf S, Hawken S, Ounpuu S (2004). on behalf of the INTERHEART Study Investigators. Effect of potentially modifiable risk factors associated with myocardial infarction in 52 countries (the INTERHEART study): case-control study. Lancet.

[17] Anand SS, Yusuf S (1997). Risk factors for cardiovascular disease in Canadians of South Asian and European origin: a pilot study of the Study of Heart Assessment and Risk in Ethnic Groups (SHARE). Clinical and investigative medicine Medecine clinique et experimentale.

[18] Giles-Corti B, Donovan RJ (2002). The relative influence of individual, social and physical environment determinants of physical activity. Soc Sci Med.

[19] Artinian NT, Fletcher GF, Mozaffarian D, Kris-Etherton P, Van Horn L, Lichtenstein AH, Kumanyika S, Kraus WE, Fleg JL, Redeker NS, et al. Interventions to promote physical activity and dietary lifestyle changes for cardiovascular risk factor reduction in adults: a scientific statement from the American Heart Association. Circulation. 122(4):406–41. 10.1161/CIR.0b013e3181e8edf1 PMC6893884 20625115

[20] Valente T (2010). Social networks and health: models, methods, applications.

[21] Berkman LF (1986). Social networks, support, and health: taking the next step forward. Am J Epidemiol.

[22] Burt RS (1987). Social Contagion and Innovation - Cohesion Versus Structural Equivalence. Am J Sociol.

[23] Fujimoto K, Unger JB, Valente TW (2012). A network method of measuring affiliation-based peer influence: assessing the influences of teammates' smoking on adolescent smoking. Child Dev.

[24] Kanaya AM, Kandula N, Herrington D, Budoff MJ, Hulley S, Vittinghoff E, Liu K (2013). Mediators of Atherosclerosis in South Asians Living in America (MASALA) Study: Objectives, Methods, and Cohort Description. Clin Cardiol.

[25] Pollard TM, Carlin LE, Bhopal R, Unwin N, White M, Fischbacher C (2003). Social networks and coronary heart disease risk factors in South Asians and Europeans in the UK. Ethnic Health.

[26] Kelly L, Patel SA, Narayan KMV, Prabhakaran D, Cunningham SA (2014). Measuring Social Networks for Medical Research in Lower-Income Settings. PLOS ONE.

[27] McPherson M, Smith-Lovin L, Brashears ME (2006). Social isolation in America: Changes in core discussion networks over two decades. Am Sociol Rev.

[28] Cornwell B, Schumm LP, Laumann EO, Graber J (2009). Social Networks in the NSHAP Study: Rationale, Measurement, and Preliminary Findings. J Gerontol B-Psychol.

[29] Valente TW, Fujimoto K, Chou CP, Spruijt-Metz D (2009). Adolescent Affiliations and Adiposity: A Social Network Analysis of Friendships and Obesity. J Adolescent Health.

[30] Bonacich P (1987). Power and Centrality: A Family of Measures. The American Journal of Sociology.

[31] Marsden PV (1987). Core discussion networks of Americans. American Sociological Review.

[32] Moore JC (1988). Self-Proxy Response Status and Survey Response Quality: A Review of the Literature. Journal of Office. Statistics.

[33] Cornwell B (2009). Network Bridging Potential in Later Life Life-Course Experiences and Social Network Position. J Aging Health.

[34] Lakon CM, Valente TW (2012). Social integration in friendship networks: The synergy of network structure and peer influence in relation to cigarette smoking among high risk adolescents. Social Science & Medicine.

[35] Kandula NR, Lauderdale DS, Baker DW (2007). Differences in self-reported health among Asians, Latinos, and non-Hispanic whites: the role of language and nativity. Ann Epidemiol.

[36] Kanaya A, Ewing S, Vittinghoff E, Herrington D, Tegeler C, Mills C, Kandula N. Acculturation and Subclinical Atherosclerosis among U.S. South Asians: Findings from the MASALA study. Journal of clinical and experimental research in cardiology. 2014;1(1) PMC4283837 25568891

[37] Marquez B, Elder JP, Arredondo EM, Madanat H, Ji M, Ayala GX (2014). Social network characteristics associated with health promoting behaviors among Latinos. Health Psychology.

[38] Marsden PV (1987). Core Discussion Networks of Americans. Am Sociol Rev.

[39] Kim HS, Sherman DK, Ko D, Taylor SE (2006). Pursuit of comfort and pursuit of harmony: Culture, relationships, and social support seeking. Pers Soc Psychol B.

[40] Phinney JS, Romero I, Nava M, Huang D (2001). The role of language, parents, and peers in ethnic identity among adolescents in immigrant families. Journal of youth and Adolescence.

[41] Rivas-Drake D, Umaña-Taylor AJ, Schaefer DR, Medina M (2017). Ethnic-Racial Identity and Friendships in Early Adolescence. Child Development.

[42] Fujimoto K, Valente TW (2013). Alcohol peer influence of participating in organized school activities: a network approach. Health Psychol.

[43] Youm Y, Laumann EO, Ferraro KF, Waite LJ, Kim HC, Park YR, Chu SH, Joo WT, Lee JA (2014). Social network properties and self-rated health in later life: comparisons from the Korean social life, health, and aging project and the national social life, health and aging project. BMC Geriatr.

[44] Litwin H (1998). Social network type and health status in a national sample of elderly Israelis. Soc Sci Med.

[45] Khosla N, Washington KT, Shaunfield S, Aslakson R (2017). Communication Challenges and Strategies of U.S. Health Professionals Caring for Seriously Ill South Asian Patients and Their Families. Journal of palliative medicine.

[46] O'Malley AJ, Marsden PV (2008). The Analysis of Social Networks. Health Serv Outcomes Res Methodol.

[47] O’Malley AJ, Arbesman S, Steiger DM, Fowler JH, Christakis NA (2012). Egocentric Social Network Structure, Health, and Pro-Social Behaviors in a National Panel Study of Americans. PLOS ONE.

[48] Hoeffel E, Rostogi S, Kim M, Shahid H.: The Asian Population: 2010. In.: U.S. Census Bureau; 2010.

