# Supplementary material for: Personal social networks and organizational affiliation of South Asians in the United States
Source: BMC Public Health. 2018 Feb 5;18:218. doi: 10.1186/s12889-018-5128-z (PMC5800071; doi:10.1186/s12889-018-5128-z)
Supplement: Additional file 1: Table S1. — Pairwise correlations of network variables, the Mediators of Atherosclerosis in South Asians Living in America (MASALA) Social Networks Study, 2014-2017. (DOCX 24 kb) [file 12889_2018_5128_MOESM1_ESM.docx]

Supplemental Table 1: Pairwise correlations of network variables, the Mediators of Atherosclerosis in South Asians Living in America (MASALA) Social Networks Study, 2014-2017

| Network measure | Network size | | Proportion Kin | | Proportion South Asian | | Proportion Female | | Proportion living in same household | | Closeness to alters (mean; 1 low to 5 high) | | | | Volume of contact with alters (contact-days/year) | | | Network density (proportion of possible alter-alter ties that exist) | | Effective size | |
| --- | --- | --- | --- | --- | --- | --- | --- | --- | --- | --- | --- | --- | --- | --- | --- | --- | --- | --- | --- | --- | --- |
| Network Size | - |  | - |  | - |  | - |  | - |  | | - | - |  | | - |  | - |  | - |  |
| Proportion Kin | -0.10 | * | - |  | - |  | - |  | - |  | | - | - |  | | - |  | - |  | - |  |
| Proportion South Asian | -0.04 |  | 0.34 | *** | - |  | - |  | - |  | | - | - |  | | - |  | - |  | - |  |
| Proportion Female | 0.00 |  | 0.11 | * | -0.04 |  | - |  | - |  | | - | - |  | | - |  | - |  | - |  |
| Proportion living in same household | -0.28 | *** | 0.42 | *** | 0.09 | * | -0.07 |  | - |  | | - | - |  | | - |  | - |  | - |  |
| Closeness to alters (mean; 1 low to 5 high) | 0.01 |  | 0.31 | *** | 0.17 | *** | 0.03 |  | 0.19 | *** | | - | - |  | | - |  | - |  | - |  |
| Volume of contact with alters (contact-days/year) | 0.44 | *** | 0.04 |  | 0.00 |  | -0.03 |  | 0.04 |  | | 0.09 |  | * | | - |  | - |  | - |  |
| Network density (proportion of possible alter-alter ties that exist) | -0.06 |  | 0.53 | *** | 0.26 | *** | -0.01 |  | 0.41 | *** | | 0.33 |  | *** | | 0.14 | ** | - |  | - |  |
| Effective size | 0.20 | *** | -0.53 | *** | -0.28 | *** | 0.02 |  | -0.42 | *** | | -0.33 |  | *** | | -0.014 |  | -0.94 | *** | - |  |
| Number of organizations affiliated with | 0.05 |  | -0.00 |  | 0.22 | *** | 0.00 |  | -0.12 | * | | 0.03 |  |  | | 0.03 |  | 0.04 |  | -0.02 |  |

* *p* < .05; ** *p* < .01; *** *p* < .001.
